# Thunderclap headache triggered by micturition: responsive to nimodipine

**DOI:** 10.1007/s10194-011-0367-8

**Published:** 2011-08-19

**Authors:** Yuan-Yuan Han, Wei Gui, Jin Zhu, Yi-Min Liu, Kai Wang, Yu Wang

**Affiliations:** 1 Department of Neurology, The First Hospital of Anhui Medical University, Jixi Road 218, Hefei, 230022 China; 2 Neuropsychology Group, Anhui Medical University Institute of Neurology, Jixi Road 218, Hefei, 230022 China

**Keywords:** Thunderclap headache, Micturition, Nimodipine, Vasospasm

## Abstract

Primary thunderclap headache (TCH) is a rare condition, of which the onset can be triggered by coughing, exercise, and sexual activity. Micturition is a recognized trigger of secondary TCH with pheochromocytoma in bladder, but not of primary TCH. We describe a patient with an apparent primary TCH, which repeatedly occurred immediately after micturition until she achieved a therapeutic dosage of nimodipine.

## Introduction

Thunderclap headache (TCH) is a hyper-acute, severe headache that reaches maximum intensity at onset. This type of headache is frequently described by patient as being ‘the worst headache of his or her life’. The term TCH was first used in the description of the headache caused by unruptured intracerebral aneurysms [1]. Because of its hyper-acute and severe characteristics, it is described as thunderclap [2]. TCH could be the conditions secondary to subarachnoid hemorrhage, unruptured intracranial aneurysm, cerebral venous sinus thrombosis, cervical artery dissection, spontaneous intracranial hypotension, acute hypertensive crisis, or a third ventricle colloid cyst [3]. But primary TCH has been described as a distinct entity when the known causes have been excluded. A primary TCH may be triggered by coughing, exercise and sexual activity and thus be classified as primary cough headache, primary exertional headache and primary headache associated with sexual activity [4]. There are also micturition-triggered TCHs though these conditions are very rare, but all the reported cases were found attributed to bladder pheochromocytoma and the headache symptom was associated with elevation of blood pressure following micturition [5, 6]. Here, we report, for the first time, a case of recurrent TCH triggered exclusively by micturition with neither pheochromocytoma in bladder nor elevation of blood pressure following micturition.

## Case report

A 52 year-old woman presented with episodes of sudden-bursting headache immediately after micturition for the last 4 days before admission to our department. She described the onset of headache as sudden, severe, and as if she had been ‘hit in the head by a car’. She characterized it as a distending headache which first occurred on the bilateral temporal region and progressively spread to all the head within 30 s. The headache reached its peak almost at onset and was rated by her as 9/10 in severity. The headache was accompanied by nausea and vomiting, but not by sense of vertigo or limb incoordination. It lasted for 1–2 h and disappeared gradually. There was no personal history of headache or other neurological disorders. She had been treated for migraine at a community hospital, but oral pain relievers failed to ease the pain.

On examination, she was alert, oriented and anxious, with a blood pressure (BP) of 108/64 mmHg, a regular heartbeat of 68 beats/minute, and a temperature of 36.8°C. Her neurological and general examination did not reveal any pathologic findings. For several times during headache attacks immediately after micturition, her BP was monitored to be in normal range.

Findings from laboratory surveys, including antinuclear antibody, C-reactive protein level, erythrocyte sedimentation rate, anticardiolipin antibody, anti-neutrophil cytoplasmic antibodies, urine tests, thyroid functions, lupus anticoagulant, serum catecholamine, were either negative or within normal limits.

Cerebrospinal fluid (CSF) examination, performed 2 h after one headache attack, had a normal opening pressure, revealed clear fluid with a leukocyte count of 3/mm^3^, a protein level of 0.39 g/l (normal range, 0.15–0.45 g/L), and a glucose level of 3.2 mmol/L (normal range, 2.5–4.4 mmol/L). CSF viral antibody test revealed negative for all the familiar virus including hepatitis B and C, cytomegalovirus, herpes simplex virus, Epstein-Barr virus.

Brain computerized tomography (CT) scan, magnetic resonance imaging (MRI), and magnetic resonance angiography (MRA) showed no signs of intracerebral hemorrhage, subarachnoid hemorrhage, infarction, thrombosis, dissection or aneurysm of the extracranial or intracranial arteries. The contrast-enhanced CT scan of the abdomen revealed no abnormalities. Electroencephalogram (EEG) was normal. Testing for anxiety and depressive disorders was conducted in the headache-free time among the attacking days, revealing a score of 16 on the Hamilton anxiety scale (HAM-A) (mild anxiety ≥14) and 9 on the Hamilton depression scale (HAM-D) (mild depression >7) [7, 8].

Antianxiety treatment with flupentixol and melitracen tablets (one tablet contains 0.5 mg flupentixol and 10 mg melitracen) at a dosage of one tablet in the morning and one tablet at noon, together with antalgesic, pregabalin, at a dosage of 150 mg daily, did not prevent the recurrence of micturitional headaches. During this time, she would micturate two to three times a day and each micturition was followed by a TCH. One week later, antianxietic and antalgesic were substituted with oral nimodipine 60 mg every 6 h and the intensity of the micturitional headaches gradually became weaker on the first day of nimodipine taking and no further micturitional headache attacks were noted on the second day. The patient was then discharged home in stable condition. Nimodipine treatment was tapered off gradually in the following 6 weeks, and no headache recurred. Six-month follow-up revealed that the patient’s condition was very well except occasional slight headaches when she met cold-wind.

## Discussion

TCH is a severe, hyper-acute headache of the sudden-bursting type like a ‘clap of thunder’, of which the pain intensity reaches a peak at onset usually within 60 s. TCH is considered to have two types: primary TCH and secondary TCH. Secondary TCH could be attributed to vascular causes including subarachnoid hemorrhage (SAH), cerebral venous sinus thrombosis (CVST), pituitary apoplexy, ischemic stroke, severe hypertension crisis, and nonvascular causes including spontaneous intracranial hypotension, third ventricle colloid cyst, intracranial infections [3]. Our MRI, MRA, and CSF examinations had excluded these possible underlying causes in this patient. In very rare situations, micturition can also trigger sudden-bursting severe headache in patients, but all these reported cases were accompanied with pheochromocytoma in bladder [6, 9, 10] and the headaches were usually associated with elevations of blood pressure following micturition [5]. The contrast-enhanced abdomen CT scan and the unelevated blood pressure during headache attack had also excluded the underlying causes of bladder pheochromocytoma. The headache in our patient seems in consistent with the presentations of primary TCH. Apart from the triggering factor micturition, the headache of our patient met the diagnostic criteria for primary TCH: (i) Severe head pain, (ii) sudden onset, reaching maximum intensity in <1 min, lasting from 1 h to 10 days, (iii) Does not recur regularly over subsequent weeks or months, and (iv) Not attributed to another disorder. Though evidence that thunderclap headache exists as a primary condition is poor, primary TCH has been described as a distinct entity [4]. A primary TCH may occur spontaneously while the patient is at rest or during light activities or may only be triggered by exertion, sexual activity, coughing and thus be classified as primary cough headache, primary exertional headache and primary headache associated with sexual activity respectively. The TCH in our patient was exclusively triggered by micturition and there was no serious underlying pathology identified to explain this TCH, thus it might be plausible to consider the micturitional headache as ‘primary’. This micturitional headache is an unusual headache not defined by the ICHD-II, therefore the reporting of this case might be helpful for the more detailed headache classification in the future.

Our patient’s response to nimodipine was dramatic and impressive during the time when headaches were still worsening. The close chronological concordance between headache relief and initiation of oral nimodipine was in favor of the direct therapeutic effect of nimodipine. Indeed, nimodipine is currently recommended in aneurysmal subarachnoid hemorrhage in order to prevent vasospasm based on its role in the relaxation of the smooth muscle of arterial blood vessel walls [11]. Thus, the recurrent micturitional TCH in our patient might be associated with cerebral vasospasm though the vasospasm was not visible by MRA. Efficacy of nimodipine was reported in primary TCH [12] as well as in primary bathing TCH [13] with vasospasm detected or non-detected. It has been suggested that primary recurrent TCH can be divided in two groups: one with diffuse vasospasm and the others without any visible vasospasm by MRA, and primary TCH, to some extent, share the same spectrum with reversible cerebral vasoconstriction syndrome (RCVS) based on their similar clinical features and the same rate of ischemic complications [14, 15]. The therapeutic efficacy of nimodipine in vasospasm associated primary TCH and RCVS as well as in our micturitional headache may suggest a similar pathogenic mechanism of vasospasm for both conditions. Unfortunately, a late control MRA or transcranial Doppler was not conducted to show any presence of late vasospasm eventually in this patient. Recently, it has been shown that, in some patients, the maximal vasospasm detected by transcranial Doppler or by MRA is respectively at 18–25 days and at 16 ± 10 days after TCH onset [16, 17]. The pathophysiological relationship between micturition and cerebral vascular change is poorly understood and speculative. There is anecdotal evidence suggesting that abdominal support, voluntary diaphragm and abdominal muscle co-activation, is necessary for effective voiding, this results in an alteration of intra-abdominal pressure. This was suggested to have causative relationship to TCH triggered by singing [18]. This intra-abdominal pressure alteration may trigger an unclear neural reflex resulting in a cerebral vascular change.

Involvement of non-organic, psychiatric factors should also be considered to contribute to the recurrent headaches, as a mild depressive (HAM-D = 16) and a mild anxiety disorder (HAM-A = 9) were revealed and a distinct phobia for micturition also found in our patient. Whereas, the negative result of the antipsychotic treatment seemly indicated that the psychiatric factors may not play a critical role in the headache recurrence. Further case reports may help to elucidate the possible role of psychiatric factors in the micturitional headache.

In summary, we treated a patient with recurrent TCH apparently triggered by micturition without any underlying organic causes, which was responsive to nimodipine dramatically. We hypothesize that the pathophysiology of this primary micturitional headache is associated with cerebral vasospasm induced by neural reflex after the intra-abdominal pressure is altered.

*Ethical standards*: The human studies have been approved by the Anhui Medical University ethics committee and have therefore been performed in accordance with the ethical standards laid down in the 1964 Declaration of Helsinki. Informed consent was obtained from the patient in this report prior to the related test and report.
